# Exercise and the Prevention of Oesophageal Cancer (EPOC) study protocol: a randomized controlled trial of exercise versus stretching in males with Barrett's oesophagus

**DOI:** 10.1186/1471-2407-10-292

**Published:** 2010-06-16

**Authors:** Brooke M Winzer, Jennifer D Paratz, Marina M Reeves, David C Whiteman

**Affiliations:** 1 The University of Queensland, School of Medicine, Burns, Trauma & Critical Care Research Centre, Brisbane QLD 4029, Australia; 2 The University of Queensland, School of Population Health, Cancer Prevention Research Centre, Brisbane QLD 4006, Australia; 3 Queensland Institute of Medical Research, Cancer Control Group, Brisbane QLD 4029, Australia

## Abstract

**Background:**

Chronic gastro-oesophageal reflux disease and excessive body fat are considered principal causes of Barrett's oesophagus (a metaplastic change in the cells lining the oesophagus) and its neoplastic progression, oesophageal adenocarcinoma. Metabolic disturbances including altered levels of obesity-related cytokines, chronic inflammation and insulin resistance have also been associated with oesophageal cancer development, especially in males. Physical activity may have the potential to abrogate metabolic disturbances in males with Barrett's oesophagus and elicit beneficial reductions in body fat and gastro-oesophageal reflux symptoms. Thus, exercise may be an effective intervention in reducing oesophageal adenocarcinoma risk. However, to date this hypothesis remains untested.

The 'Exercise and the Prevention of Oesophageal Cancer Study' will determine whether 24 weeks of exercise training will lead to alterations in risk factors or biomarkers for oesophageal adenocarcinoma in males with Barrett's oesophagus. Our primary outcomes are serum concentrations of leptin, adiponectin, tumour necrosis factor-alpha, C-reactive protein and interleukin-6 as well as insulin resistance. Body composition, gastro-oesophageal reflux disease symptoms, cardiovascular fitness and muscular strength will also be assessed as secondary outcomes.

**Methods/Design:**

A randomized controlled trial of 80 overweight or obese, inactive males with Barrett's oesophagus will be conducted in Brisbane, Australia. Participants will be randomized to an intervention arm (60 minutes of moderate-intensity aerobic and resistance training, five days per week) or a control arm (45 minutes of stretching, five days per week) for 24 weeks. Primary and secondary endpoints will be measured at baseline (week 0), midpoint (week 12) and at the end of the intervention (week 24).

**Discussion:**

Due to the increasing incidence and very high mortality associated with oesophageal adenocarcinoma, interventions effective in preventing the progression of Barrett's oesophagus are urgently needed. We propose that exercise may be successful in reducing oesophageal adenocarcinoma risk. This primary prevention trial will also provide information on whether the protective association between physical activity and cancer is causal.

**Trial Registration:**

ACTRN12609000401257

## Background

**Figure 1:**
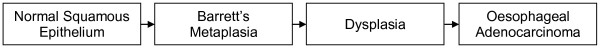
**The Barrett's metaplasia-dysplasia-adenocarcinoma sequence**.

During the past three decades, the incidence of oesophageal adenocarcinoma (AC) has risen by more than 300% in females and 500% in males; faster than any other cancer in Western Europe, America and Australia [1-5]. Moreover, the incidence of Barrett's oesophagus, the metaplastic precursor to oesophageal AC (Figure 1), is also rising [6]. The prognosis for patients with AC is poor with a 5 year survival rate of 10-15% [7,8].

Chronic reflux of gastric contents into the lower oesophagus is widely accepted as the primary cause of Barrett's oesophagus and oesophageal AC [9]. Recent research has demonstrated that excessive body fat is also a cause of oesophageal AC and is likely to act through promoting neoplastic progression from Barrett's oesophagus [10]. Obesity [body mass index (BMI) > 30 kg/m^2^] is a determinant of acid reflux [11], and has been associated with a two-fold increase in the risk of Barrett's oesophagus [12] and up to a three-fold increase in oesophageal AC risk [13,14]. Interestingly, the co-occurrence of obesity and frequent reflux symptoms increases a person's risk of Barrett's oesophagus by over 30-fold [12]. Body fat distribution also influences Barrett's oesophagus risk. Central obesity indicated by a waist circumference of > 80 cm has been shown to increase the risk of Barrett's oesophagus by over two-fold, independent of BMI and gastro-oesophageal reflux [15,16].

Adipose tissue is a dynamic endocrine organ. Adipocytes secrete numerous hormones or 'adipokines' that exhibit mitogenic activity such as leptin, adiponectin, interleukin-6 (IL-6) and tumour necrosis factor-alpha (TNF-α). It has been postulated that these hormones may mediate the progression of Barrett's oesophagus to cancer (Table 1).

**Table 1:** Biomarkers associated with the progression of Barrett's oesophagus to oesophageal adenocarcinoma.

Biomarker	Direction	Putative mechanism of promoting oesophageal adenocarcinoma
Gastro-oesophageal reflux frequency and severity	Increased	Chronic inflammation and damage to oesophageal epithelium
Central obesity	Increased	Systemic metabolic dysfunction Increased reflux of gastric acid into the lower oesophagus via increased intra-abdominal pressure and/or hiatus hernia
Leptin	Increased	Mitogenic Angiogenic Anti-apoptotic
Adiponectin	Decreased	Increased insulin resistance Pro-inflammatory Anti-apoptotic
Inflammatory mediators:	Increased	Mitogenic Angiogenic
C-reactive protein		Increased differentiation
Tumour necrosis factor-α		Anti-apoptotic
Interleukin-6		Decreased DNA repair
Insulin	Increased	Mitogenic Anti-apoptotic Increased leptin Increased tumour necrosis factor-α Decreased adiponectin

### Leptin

The adipokine leptin is secreted by adipocytes and gastric chief cells and is positively associated with insulin levels, inflammation and body fat. *In vitro*, leptin has been shown to elicit mitogenic [17-19], angiogenic [17,18,20] and anti-apoptotic [18,19] effects when administered to oesophageal AC cell lines, enhancing cellular proliferation. Leptin receptors are also present on oesophageal epithelial cells providing a pathway for signalling [21]. A recent epidemiological study reported that male patients with Barrett's oesophagus had significantly higher leptin levels than BMI-matched controls, but no such association was seen for women [22]. These findings implicate leptin as a likely candidate mediator driving the progression from Barrett's oesophagus to oesophageal AC.

### Adiponectin

Adiponectin is another adipokine implicated in the biochemical pathways to oesophageal AC development. Unlike leptin, adiponectin is inversely related to obesity and has anti-inflammatory and insulin sensitising properties. Adiponectin circulates as three oligomeric isoforms: high molecular weight (HMW), medium molecular weight (MMW) and low molecular weight (LMW) adiponectin. HMW adiponectin may be the major bioactive form as decreased levels are more closely correlated with insulin resistance and metabolic dysfunction than total adiponectin [23].

*In vitro*, total adiponectin has been shown to act on specific receptors to increase apoptosis [24] and inhibit leptin-induced proliferation in oesophageal AC cell lines [20]. The expression of adiponectin receptors have also been shown to be reduced in Barrett's oesophagus epithelium at the mRNA level [24]. Low levels of total adiponectin have been associated with Barrett's oesophagus [25], oesophageal AC [26], and a range of other obesity-related cancers [27].

### Systemic Inflammation

Individuals with Barrett's oesophagus also exhibit signs of chronic inflammation indicated by higher circulating levels of inflammatory mediators such as IL-6 [28,29] and TNF-α [30]. The protein expression of TNF-α is also notably elevated in Barrett's oesophagus tissue samples and even more so in AC tissue [31]. Additionally, TNF-α has been shown to stimulate the proliferation of oesophageal AC cell lines *in vitro *[31]. Further evidence of a link between inflammation and oesophageal AC comes from findings that weekly users of aspirin had a 50% reduction in oesophageal AC risk [32-34].

### Insulin Resistance

Because insulin has a range of actions (mitogenic and anti-apoptotic) in addition to its effect on blood glucose concentrations, it has been postulated that hyperinsulinaemia (secondary to insulin resistance) may also drive proliferative and metaplastic changes in gastrointestinal mucosa [35]. Furthermore, elevated levels of leptin and inflammatory mediators such as TNF-α, are also characteristics of an insulin-resistant state [36].

### Exercise may Modulate Oesophageal Cancer Risk

Due to the increasing incidence and very high mortality associated with oesophageal AC, interventions effective in preventing the neoplastic progression of Barrett's oesophagus are urgently needed. Although limited, there are some epidemiological data to suggest that exercise may modulate oesophageal AC risk. Data from one large cohort study suggests that ≥ 100 minutes of physical activity per week (measured by self report), compared to no physical activity is associated with a significant 32% reduction in AC risk, although this association was attenuated when further adjustment was made for BMI [37]. Higher levels of self reported occupational physical activity before the age of 65 have also been associated with a 39% decreased risk of oesophageal AC in one population-based case-control study [38].

Physical activity may act to modulate oesophageal AC risk by reversing the metabolic aberrations associated with Barrett's oesophagus and oesophageal AC. Evidence from clinical trials in overweight individuals without Barrett's oesophagus, suggests that physical activity can significantly reduce leptin concentrations with [39-41] and without [42-44] accompanying exercise induced weight-loss. Beneficial increases in adiponectin have also been observed following 4 weeks of exercise, with results remaining significant after adjusting for body weight, body fat and plasma insulin [45]. Furthermore, physical activity is considered to play an integral role in the treatment and prevention of insulin resistance [46] and has anti-inflammatory effects [47,48] which may also benefit Barrett's oesophagus patients. Finally, exercise may lead to reductions in centrally stored body fat which may subsequently reduce gastro-oesophageal reflux symptoms, contributing further to reductions in oesophageal AC risk.

Overall, preliminary data from epidemiological studies and evidence from clinical trials in overweight populations provide a strong rationale for trialing physical activity interventions in patients with Barrett's oesophagus to reduce cancer risk. To date no clinical trials have tested the hypothesis that physical activity reduces oesophageal AC risk factors in males with Barrett's oesophagus.

The aim of the "Exercise and the Prevention of Oesophageal Cancer" (EPOC) study is to determine the effect of a 24 week moderate-intensity exercise intervention versus stretching on biomarkers associated with oesophageal AC development in overweight or obese, inactive males with Barrett's oesophagus. Our primary outcomes are serum concentrations of leptin, adiponectin, TNF-α, C-reactive protein (CRP), IL-6 and insulin resistance. Secondary outcomes include body composition, gastro-oesophageal reflux symptoms, cardiovascular fitness and muscular strength.

## Methods/Design

**Figure 2:**
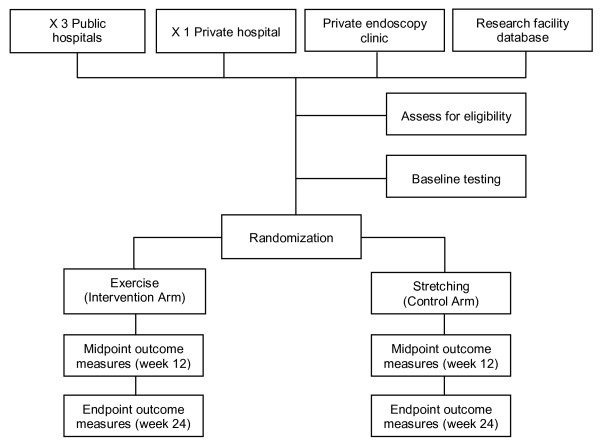
**CONSORT diagram**.

EPOC is a randomized controlled trial with participants randomized into a 24-week exercise intervention arm or a control arm (Figure 2). All outcomes will be measured by study personnel blinded to participants' group allocation. The study is being conducted in Brisbane, Australia. The protocol has been approved by The University of Queensland Human Ethics Committee, and the Human Ethics Committees of the four participating hospitals - Royal Brisbane & Women's Hospital, Princess Alexandra Hospital, Prince Charles Hospital and The Wesley Hospital.

### Participants

This study is aiming to recruit 80 males with Barrett's oesophagus. Barrett's oesophagus is defined as the abnormal appearance of the lining of the distal oesophagus (determined via endoscopy) in addition to histological evidence of intestinal metaplasia (determined via biopsy) [49]. Both newly diagnosed and prevalent cases of Barrett's oesophagus will be included. The study is limited to males only as males are twice as likely to develop oesophageal AC than females [50]. Participants must be English speaking, residing in greater Brisbane, aged 18-70 years, have a BMI between 25 kg/m^2 ^and 34.99 kg/m^2 ^and be performing ≤ 60 minutes per week of moderate-vigorous intensity exercise during the previous 6 weeks. Additionally, participants must not have gained or lost ≥ 5 kg of body weight during the past 6 months. Exclusion criteria include cardiac, respiratory, renal, liver, neurological or inflammatory disease. Individuals with diabetes mellitus, hypertension, hypotension, a cancer diagnosis within 5 years or orthopaedic injuries limiting exercise participation, will also be excluded.

### Recruitment

Participants will be identified and recruited through gastroenterology department appointments, hospital databases and a research study database. Recruitment will take place over a period of 15 months (May 2009 - August 2010). Patients attending appointments at the gastroenterology departments of three large tertiary hospitals, one private hospital and a private gastroenterology clinic in Brisbane, Australia, are provided with an information sheet by their treating doctor and given an expression of interest form to complete. Potential participants identified by hospital personnel from hospital databases will be posted a letter and an expression of interest form. Potential participants who have expressed interest in joining the study will then be contacted by telephone by study personnel to have the study explained and be screened for eligibility. Participants will enter the study once written informed consent has been obtained.

### Randomization

Participants are allocated to the intervention arm or the control arm via computerised randomization http://www.randomization.com, following completion of baseline assessment. The randomization sequence was generated by a research assistant not involved with the study. Group allocation is concealed from investigators using sealed, numbered envelopes.

### Intervention Arm

Participants randomized to the intervention arm of the study undertake 60 minutes of exercise, five days per week for 24 weeks. Each session involves: 30 minutes of moderate-intensity aerobic training (60-70% of maximum heart rate) and 30 minutes of resistance training (plus a 5 minute warm-up and cool-down of light intensity). Participants perform one exercise session per week at a hospital gymnasium in small groups under the supervision of study personnel (physiotherapist/exercise physiologist). In addition, each participant is provided with a free gym membership to complete another four exercise sessions independently at a privately run health club in their local area.

Participants perform aerobic exercise on treadmills, cycling or rowing stationary ergometers and elliptical machines. During the sessions exercise intensity is measured via the unmodified (6-20) BORG scale. Participants are instructed to maintain their rate of perceived exertion between "somewhat hard" and "hard" (13-15) [51]. The resistance component consists of performing one set of 12-15 repetitions during weeks 0-8; and two sets of 8-10 repetitions during weeks 9-24. Resistance is prescribed to achieve muscular fatigue between 8-15 repetitions depending on the training phase. Exercises include: chest press, leg press, shoulder press, seated row, lunges, assisted chin up, assisted dip and core stability.

The program is consistent with the current World Cancer Research Fund and American Institute for Cancer Research recommendations for cancer prevention which advocate 30-60 minutes of moderate-intensity exercise on most if not all days of the week [10].

### Control Arm

Participants allocated to the control arm attend a hospital gymnasium once a week to perform 45 minutes of stretching in small groups under the supervision of study personnel. They are also instructed to perform the stretching program independently at home, four times per week. Stretching will act as an 'attention' control condition. Participants in the control arm are instructed not to commence a new exercise program during the study. At the conclusion of the study they are offered an exercise program and a complimentary three month gym membership to a private health club in their local area.

### Attendance and Adherence

Participants document the details of their independent exercise or stretching sessions daily in a physical activity diary. For participants in the intervention arm, attendance at the private health club is also electronically recorded each time they visit via their membership card. Secondary outcome measures such as cardiovascular fitness, muscular strength and body composition will also provide information regarding adherence to the protocol.

### Primary Outcomes

All primary outcomes are measured at baseline (week 0), midpoint (week 12) and at the conclusion of the intervention (week 24). Biomarkers associated with oesophageal AC development will be measured as primary outcomes via blood samples. Fasting blood samples of 30 ml will be obtained by blood collectors from an accredited pathology laboratory from the antecubital vein between 08:00-10:00 am and at least 24 hours post exercise to minimize diurnal fluctuations and acute exercise effects. Blood samples will be centrifuged within 20 minutes of collection and stored at -80°C. Laboratory personnel will analyse the samples in two batches over a period of 24 months.

Serum leptin will be analysed via radioimmunoassay (RIA) (Linco Research, Missouri, USA) and serum adiponectin (total and HMW) by an enzyme-linked immunosorbent assay (ELISA) (Linco Research, Missouri, USA). The inter- and intra-assay coefficients of variation (CV) are < 8% for leptin and < 9% for adiponectin.

Fasting serum insulin will be analysed using an immunoenzymatic 'sandwich' assay via an ACCESS system (Beckman Coulter, Fullerton, USA) and plasma glucose concentrations determined with an oxygen rate method via the SYNCHRON system (Beckman Coulter, Fullerton, USA) with an inter- and intra-assay CV of < 5% for both insulin and glucose. Insulin resistance will be calculated using the reciprocal index of homeostasis model assessment (HOMA-IR) [52] and the Quantitative Insulin Sensitivity Check Index (QUICKI) [53]. Both measures calculate ratios of fasting insulin:glucose concentrations using different algorithms and have been shown to be reproducible and valid measures of insulin resistance.

Serum concentrations of CRP will be analysed using a high sensitivity Near Infrared Particle Immunoassay with the SYNCHRON system (Beckman Coulter, Fullerton, USA). Inter- and intra-assay CV is < 5%. IL-6 will be measured in serum with the Immulite 2000 analyser using an immunometric assay (Siemens, USA) with an inter- and intra-assay CV of < 7%. Serum TNF-α will be measured using a Bio-Plex suspension array system via immunoassay (Bio-Rad laboratories, Hercules, USA), with inter-assay CV of < 8% and intra-assay CV of < 6%.

### Secondary Outcomes

Secondary outcomes will also be measured at week 0, 12 and 24.

#### Body Composition

Body fat and fat free mass will be measured in triplicate using bioimpedance spectroscopy (ImpediMed SFB7, ImpediMed Ltd., Australia). Typical coefficients of variation within a measurement session range from 0.3 to 3.0% [54]. Waist circumference will be calculated in duplicate at the midpoint between the lower costal (rib) border and the iliac crest. Hip circumference will be measured in duplicate as the maximum diameter at the greater trochanter. Waist-to-hip ratio will also be determined.

BMI will be calculated (weight [kg]/height [m]^2^) as it is a reasonable measure of global adiposity. Height is measured using a stadiometer to 1 mm, without shoes, arms by side and heels together. Weight is measured to the nearest 0.1 kg in light clothing, without shoes, on a calibrated scale (UC-321, A&D Co. Ltd., Japan) with an empty bladder.

#### Cardiovascular Fitness

Cardiovascular fitness will be estimated using peak oxygen uptake (VO_2peak_) during the Modified Shuttle Walk Test [55]. VO_2peak _will be measured using a Cortex Metamax 3 portable metabolic analyser (Cortex:biophysik, GMbH, Germany) during the test. VO_2peak _is the highest oxygen uptake achieved with an increasing external work rate, but unlike VO_2max_, a plateau in oxygen uptake may not be observed. VO_2peak _is a useful estimation of cardiovascular fitness as not every participant may reach VO_2max _before symptom limitation occurs [56].

#### Muscle Strength

One-repetition maximum (1 RM) tests will be performed on the bench press and leg press to measure muscle strength. 1 RM is the maximum weight a person can lift only one time with correct technique. 1 RM has shown to be a reliable and valid measure of strength in middle aged, untrained adults [57].

#### Gastro-oesophageal Reflux Disease

There is no gold standard test to diagnose or monitor gastro-oesophageal reflux disease [58]. Gastro-oesophageal reflux symptoms will be measured using the Gastro-esophageal Reflux Disease Impact Scale (GIS) which is short and simple to use; responsive to change over time; completed by patients and has documented internal consistency, reproducibility and construct validity [59].

### Monitoring Confounding Variables

Participants will document medication use and all protocol and non-protocol exercise performed in a daily log; smoking habits will also be recorded every 4 weeks. The International Physical Activity Questionnaire (IPAQ), (long, last 7 days, self-administered format) [60,61] will also be completed by participants at week 0, 12 and 24 and will provide additional information regarding incidental, job and leisure time physical activity; and sitting time over a one week period. All participants will be instructed to maintain a consistent diet throughout the study and this will be evaluated via a Food Frequency Questionnaire completed at week 0, 12 and 24.

### Sample Size Calculation

There is limited evidence on which to base the minimum detectable difference for the sample size calculation. Namely, the magnitude of change in the primary biomarker outcomes that is associated with a reduction in oesophageal AC risk is not known. A minimum difference of 10% between the means of the intervention arm and the control arm for all primary outcomes is likely to produce a clinical benefit and is achievable by the intervention. Standard deviations were based on those observed in previous clinical trials investigating the effect of exercise on overweight or obese, inactive, healthy males. Thus, a sample size of 40 participants per group (80 in total) is needed to detect a minimal difference of 10% in primary outcomes between groups, with 80% power, a type I error of 5% and allowing for 10% attrition (two-tailed).

### Statistical Analysis

Generalised linear models, estimating variance appropriately for repeated measures, will be used to determine whether there are differences between the exercise intervention group and the control group in primary and secondary outcomes. Models will be adjusted for baseline outcome values, to account for regression to the mean, as well as the main effects of group, time and their interaction. Mediation and moderation of intervention effects will be examined with change in body weight, body fat, minutes exercised per week and VO_2peak _considered a priori to be of potential interest. Analyses will be performed using intention-to-treat principles and on a per-protocol basis. Statistical significance will be set at p < 0.05 (two-tailed).

## Discussion

The EPOC study aims to determine whether exercise can modulate the biochemical pathways to oesophageal cancer development in males with the pre-malignant condition Barrett's oesophagus. Exercise may prove to be an effective intervention in reducing the risk of oesophageal AC development which would have important health implications for males with Barrett's oesophagus.

Moreover, findings from the EPOC study will help to further define the role of exercise in the primary prevention of cancer by adding to the limited number of clinical trials of exercise interventions and cancer-related biomarkers [62-71]. As participants targeted by the EPOC study may be at an even higher cancer risk than those previously studied, greater exercise intervention effects may be observed. Lastly, the biological mechanisms underlying the inverse association between physical activity and carcinogenesis can be further explored.

## Competing interests

The authors declare that they have no competing interests.

## Authors' contributions

DCW developed the study concept. BMW, DCW and JDP developed the study protocol. BMW drafted the manuscript. All authors contributed to the final manuscript.

## Pre-publication history

The pre-publication history for this paper can be accessed here:

http://www.biomedcentral.com/1471-2407/10/292/prepub

## References

[B1] Blot WJ Devesa SS Kneller RW Rising incidence of adenocarcinoma of the esophagus and gastric cardia JAMA 1991 265 1287 1296 10.1001/jama.265.10.1287 1995976

[B2] Lord RV Law MG Ward RL Giles GG Thomas RJ Thursfield V Rising incidence of oesophageal adenocarcinoma in men in Australia J Gastroenterol Hepatol 1998 13 4 356 362 10.1111/j.1440-1746.1998.tb00646.x 9641297

[B3] Everhart JE Ruhl CE Burden of digestive diseases in the United States Part I: Overall and upper gastrointestinal diseases Gastroenterology 2009 136 376 386 10.1053/j.gastro.2008.12.015 19124023

[B4] Vizcaino AP Moreno V Lambert R Parkin DM Time trends incidence of both major histologic types of esophageal carcinomas in selected countries, 1973-1995 Int J Cancer 2002 99 860 868 10.1002/ijc.10427 12115489

[B5] Pohl H Welch HG The role of overdiagnosis and reclassification in the marked increase of esophageal adenocarcinoma incidence J Natl Cancer Inst 2005 97 2 142 146 10.1093/jnci/dji024 15657344

[B6] Kendall BJ Whiteman DC Temporal changes in the endoscopic frequency of new cases of Barrett's oesophagus in an Australian health region Am J Gastroenterol 2006 101 1178 1182 10.1111/j.1572-0241.2006.00548.x 16771933

[B7] Parkin DM Bray F Ferlay J Pisani P Global cancer statistics 2002 CA Cancer J Clin 2005 55 74 108 10.3322/canjclin.55.2.74 15761078

[B8] Polednak AP Trends in survival for both histological types of esophageal cancer in US Surveillance, Epidemiology and End Results areas Int J Cancer 2003 105 98 100 10.1002/ijc.11029 12672037

[B9] Lagergren J Bergstrom R Lindgren A Nyren O Symptomatic gastroesophageal reflux as a risk factor for esophageal adenocarcinoma N Engl J Med 1999 340 825 831 10.1056/NEJM199903183401101 10080844

[B10] World Cancer Research Fund/American Institute for Cancer Research The second expert report: food, nutrition, physical activity and the prevention of cancer: a global perspective 2007 Washington DC: American Institute for Cancer Research

[B11] Pandolfino JE El-Serag HB Zhang Q Shah N Ghosh SK Kahrilas PJ Obesity: a challenge to esophagogastric junction integrity Gastroenterology 2006 130 3 639 649 10.1053/j.gastro.2005.12.016 16530504

[B12] Smith KJ O'Brien SM Smithers M Gotley DC Webb PM Green A Whiteman DC Interactions among smoking, obesity and symptoms of acid reflux in Barrett's oesophagus Cancer Epidemiol Biomarkers Prev 2005 14 2481 2486 10.1158/1055-9965.EPI-05-0370 16284367 PMC1481636

[B13] Hampel H Abraham NS El-Serag HB Meta-analysis: obesity and the risk for gastroesophageal reflux disease and its complications Ann Intern Med 2005 143 199 211 16061918 10.7326/0003-4819-143-3-200508020-00006

[B14] Whiteman DC Sadeghi S Pandeya N Smithers BM Gotley DC Bain CJ Webb PM Green AC Combined effects of obesity, acid reflux and smoking on the risk of adenocarcinomas of the oesophagus Gut 2008 57 173 180 10.1136/gut.2007.131375 17932103

[B15] Edelstein ZR Farrow DC Bronner MP Rosen SN Vaughan TL Central adiposity and risk of Barrett's esophagus Gastroenterology 2007 133 403 411 10.1053/j.gastro.2007.05.026 17681161

[B16] Corley DA Kubo A Levin TR Block G Habel L Zhao W Leighton P Quesenberry C Rumore GJ Buffler PA Abdominal obesity and body mass index as risk factors for Barrett's esophagus Gastroenterology 2007 133 34 41 10.1053/j.gastro.2007.04.046 17631128

[B17] Somasundar P Riggs D Jacksona B Vona-Davis L McFadden DW Leptin stimulates esophageal adenocarcinoma growth by nonapoptotic mechanisms Am J Surg 2003 186 575 578 10.1016/j.amjsurg.2003.07.017 14599628

[B18] Beales IL Ogunwobi OO Leptin synergistically enhances the anti-apoptotic and growth-promoting effects of acid in OE33 oesophageal adenocarcinoma cells in culture Mol Cell Endocrinol 2007 274 1-2 60 68 10.1016/j.mce.2007.05.017 17618045

[B19] Ogunwobi O Mutungi G Beales IL Leptin stimulates proliferation and inhibits apoptosis in Barrett's oesophageal adenocarcinoma cells by cyclooxygenase-2-dependent, prostaglandin-E2-mediated transactivation of the epidermal growth factor receptor and c-Jun NH2-terminal kinase activation Endocrinology 2006 147 4505 4516 10.1210/en.2006-0224 16740977

[B20] Ogunwobi OO Beales IL Globular adiponectin, acting via adiponectin receptor-1, inhibits leptin-stimulated oesophageal adenocarcinoma cell proliferation Mol Cell Endocrinol 2008 285 1-2 43 50 10.1016/j.mce.2008.01.023 18313838

[B21] Francois F Roper J Goodman AJ Pei Z Ghumman M Mourad M de Perez AZ Perez-Perez GI Tseng CH Blaser MJ The association of gastric leptin with oesophageal inflammation and metaplasia Gut 2008 57 1 16 24 10.1136/gut.2007.131672 17761783

[B22] Kendall BJ Macdonald GA Hayward NK Prins JB Brown I Walker N Pandeya N Green AC Webb PM Whiteman DC Leptin and the risk of Barrett's oesophagus Gut 2008 57 448 454 10.1136/gut.2007.131243 18178609

[B23] Wang Y Lam KSL Yau M-h Xu A Post-translational modifications of adiponectin: mechanisms and functional implications Biochem J 2008 409 3 623 633 10.1042/BJ20071492 18177270

[B24] Konturek PC Burnat G Rau T Hahn EG Konturek S Effect of adiponectin and ghrelin on apoptosis of Barrett adenocarcinoma cell line Dig Dis Sci 2008 53 3 597 605 10.1007/s10620-007-9922-1 17763959

[B25] Rubenstein JH Dahlkemper A Kao JY Zhang M Morgenstern H McMahon L Inadomi JM A pilot study of the association of low plasma adiponectin and Barrett's esophagus Am J Gastroenterol 2008 103 6 1358 1364 10.1111/j.1572-0241.2008.01823.x 18510610

[B26] Yildirim A Bilici M Cayir K Yanmaz V Yildirim S Tekin SB Serum adiponectin levels in patients with esophageal cancer Jpn J Clin Oncol 2009 39 2 92 96 10.1093/jjco/hyn143 19116211

[B27] Kelesidis I Kelesidis T Mantzoros CS Adiponectin and cancer: a systematic review Br J Cancer 2006 94 1221 1225 10.1038/sj.bjc.6603051 16570048 PMC2361397

[B28] Moons LMG Kusters JG Bultman E Kuipers EJ Van Dekken H Tra WMW Kleinjan A Kwekkeboom J van Vliet AHM Siersema PD Barrett's oesophagus is characterised by a predominantly humoral inflammatory response J Pathol 2005 207 269 276 10.1002/path.1847 16177953

[B29] Dvorakova K Payne C Ramsey L Increased expression and secretion of interleukin-6 in patients with Barrett's esophagus Clin Cancer Res 2004 10 2020 2028 10.1158/1078-0432.CCR-0437-03 15041721

[B30] Eksteen JA Scott PA Perry I Jankowski JA Inflammation promotes Barrett's metaplasia and cancer: a unique role for TNF-alpha Eur J Cancer Prev 2001 10 163 166 10.1097/00008469-200104000-00008 11330458

[B31] Tselepis C Perry I Dawson C Hardy R Darnton SJ McConkey C Stuart RC Wright N Harrison R Jankowski JA Tumour necrosis factor-a in Barrett's oesophagus: a potential novel mechanism of action Oncogene 2002 21 6071 6081 10.1038/sj.onc.1205731 12203119

[B32] Sadeghi S Bain CJ Pandeya N Webb PM Green A Whiteman DC Aspirin, nonsteroidal anti-inflammatory drugs, and the risks of cancers of the esophagus Cancer Epidemiol Biomarkers Prev 2008 17 5 1 10 10.1158/1055-9965.EPI-07-2852 18483339

[B33] Vaughan TL Dong LM Blount PL Ayub K Odze R Sanchez CA Rabinovitch PS Reid BJ Non-steroidal anti-inflammatory drugs and risk of neoplastic progression in Barrett's oesophagus: a prospective study Lancet Oncology 2005 6 945 952 10.1016/S1470-2045(05)70431-9 16321762

[B34] Corley DA Kerlikowske K Verma R Buffler P Protective association of aspirin/NSAIDs and esophageal cancer: A systematic review and meta-analysis Gastrenterol 2003 124 47 56 10.1053/gast.2003.50008 12512029

[B35] Komninou D Ayonote A Richie JP Jr Rigas B Insulin resistance and its contribution to colon carcinogenesis Exp Biol Med (Maywood) 2003 228 4 396 405 12671184 10.1177/153537020322800410

[B36] Boyd DB Insulin and cancer Integr Cancer Ther 2003 2 4 315 329 10.1177/1534735403259152 14713323

[B37] Leitzmann MF Koebnick C Freedman ND Park Y Ballard-Barbash R Hollenbeck A Schatzkin A Abnet CC Physical activity and esophageal and gastric carcinoma in a large prospective study Am J Prev Med 2009 36 2 112 119 10.1016/j.amepre.2008.09.033 19062237 PMC2655147

[B38] Vigen C Bernstein L Wu AH Occupational physical activity and risk of adenocarcinomas of the esophagus and stomach Int J Cancer 2006 118 4 1004 1009 10.1002/ijc.21419 16152595

[B39] Perusse L Collier G Gagnon J Leon AS Rao DC Skinner JS Wilmore JH Nadeau A Zimmet PZ Bouchard C Acute and chronic effects of exercise on leptin levels in humans J Appl Physiol 1997 83 1 5 10 9216937 10.1152/jappl.1997.83.1.5

[B40] Fatouros IG Tournis S Leontsini D Jamurtas AZ Sxina M Thomakos P Manousaki M Douroudos I Taxildaris K Mitrakou A Leptin and adiponectin responses in overweight inactive elderly following resistance training and detraining are intensity related J Clin Endocrinol Metab 2005 90 11 5970 5977 10.1210/jc.2005-0261 16091494

[B41] Thong FSL Hudson R Ross R Janssen I Graham TE Plasma leptin in moderately obese men: independent effects of weight loss and aerobic exercise Am J Physiol-Endocrinol Metab 2000 279 2 E307 E313 10913030 10.1152/ajpendo.2000.279.2.E307

[B42] Reseland JE Anderssen SA Solvoll K Hjermann I Urdal P Holme I Drevon CA Effect of long-term changes in diet and exercise on plasma leptin concentrations Am J Clin Nutrition 2001 73 2 240 245 10.1093/ajcn/73.2.240 11157319

[B43] Pasman WJ Westerterp-Plantenga MS Saris WH The effect of exercise training on leptin levels in obese males Am J Physiol 1998 274 2 E280 286 9486159 10.1152/ajpendo.1998.274.2.E280

[B44] Ishii T Yamakita T Yamagami K Yamamoto T Miyamoto m Koichi Kawasaki Masayuki Hosoi Katsunobu Yoshioka Toshihiko Sato Shiro Tanaka Effect of exercise training on serum leptin levels in type 2 diabetic patients Metabolism 2001 50 10 1136 1140 10.1053/meta.2001.26745 11586483

[B45] Bluher M Bullen JW Lee JH Kralisch S Fasshauer M Kloting N Niebauer J Schon MR Williams CJ Mantzoros CS Circulating adiponectin and expression of adiponectin receptors in human skeletal muscle: Associations with metabolic parameters and insulin resistance and regulation by physical training J Clin Endocrinol Metab 2006 91 6 2310 2316 10.1210/jc.2005-2556 16551730

[B46] Borghouts LB Keizer HA Exercise and insulin sensitivity: A review Int J Sports Med 2000 21 1 1 12 10.1055/s-2000-8847 10683091

[B47] Lakka TA Lakka H-M Rankinen T Leon AS Rao DC Skinner JS Wilmore JH Bouchard C Effect of exercise training on plasma levels of C-reactive protein in healthy adults: the HERITAGE Family Study Eur Heart J 2005 26 19 2018 2025 10.1093/eurheartj/ehi394 15987707

[B48] Milani RV Lavie CJ Mehra MR Reduction in C-reactive protein through cardiac rehabilitation and exercise training J Am Coll Cardiol 2004 43 1056 1061 10.1016/j.jacc.2003.10.041 15028366

[B49] Sharma P McQuaid K Dent J Fennerty B Sampliner RE Spechler SJ Cameron AJ Corley DA Falk GW Goldblum J A critical review of the diagnosis and management of Barrett's esophagus:The AGA Chicago Workshop Gastrenterology 2004 127 310 330 10.1053/j.gastro.2004.04.010 15236196

[B50] Yousef F Cardwell C Cantwell MM Galway K Johnston BT Murray L The incidence of esophageal cancer and high grade dysplasia in Barrett's esophagus: A systematic review and meta-analysis Am J Epidemiol 2008 168 237 249 10.1093/aje/kwn121 18550563

[B51] Borg G Perceived exertion as an indicator of somatic stress Scand J Rehabil Med 1970 2 92 99 5523831

[B52] Mathews DR Hosker JR Rudenski AS Naylor BA Treacher DF Turner RC Homeostasis model assessment: insulin resistance and beta-cell function from fasting plasma glucose and insulin concentrations in man Diabetologia 1985 28 412 121 10.1007/BF00280883 3899825

[B53] Katz A Nambi SS Mather K Baron AD Follmann DA Sullivan G Quon MJ Quantitative insulin sensitivity check index: a simple, accurate method for assessing insulin sensitivity in humans J Clin Endocrinol Metab 2000 85 2402 2410 10.1210/jc.85.7.2402 10902785

[B54] Cornish B The Evaluation of multiple frequency bioelectrical impedance analysis for the assessment of body water volumes in healthy humans Eur J Clin Nutr 1996 50 159 164 8654329

[B55] Singh S Morgan MD Scott S Walters D Hardman AE Development of a shuttle walking test of disability in patients with chronic airways obstruction Thorax 1992 47 1019 1024 10.1136/thx.47.12.1019 1494764 PMC1021093

[B56] Fletcher G Exercise standards for testing and training: a statement for healthcare professionals from the American heart Association Circulation 2001 104 1694 1740 10.1161/hc3901.095960 11581152

[B57] Levinger I Goodman C Hare DL Jerums G Toia D Selig S The reliability of the 1RM strength test for untrained, middle-aged individuals J Sci Med in Sport 2009 12 310 316 10.1016/j.jsams.2007.10.007 18078784

[B58] Moayyedi P Talley NJ Gastro-oesophageal reflux disease Lancet 2006 367 2086 2100 10.1016/S0140-6736(06)68932-0 16798392

[B59] Jones R Coyne K Wiklund I The Gastro-oesophageal Reflux Disease Impact Scale: a patient management tool for primary care Aliment Pharmacol Ther 2007 25 12 1451 1459 17539985 10.1111/j.1365-2036.2007.03343.x

[B60] Hallal PC Victora CG Reliability and validity of the International Physical Activity Questionnaire (IPAQ) Med Sci Sports Exerc 2004 36 556 10.1249/01.MSS.0000117161.66394.07 15076800

[B61] Booth ML Assessment of physical activity: an international perspective Res Q Exercise Sport 2000 71 2 s114 120 10925833

[B62] Abrahamson PE King IB Ulrich CM Rudolph RE Irwin ML Yasui Y Surawicz C Lampe JW Lampe PD Morgan A No effect of exercise on colon mucosal prostaglandin concentrations: A 12-month randomized controlled trial Cancer Epidemiol Biomarkers Prev 2007 16 11 2351 2356 10.1158/1055-9965.EPI-07-0120 18006923

[B63] Campbell KL McTiernan A Li SS Sorensen B Yasui Y Lampe JW King IB Ulrich C Rudolph R Irwin M Effect of a 12-month exercise intervention on the apoptotic regulating proteins Bax and Bcl-2 in colon crypts: a randomized controlled trial Cancer Epidemiol Biomarkers Prev 2007 16 9 1767 1774 10.1158/1055-9965.EPI-07-0291 17855695

[B64] McTiernan A Yasui Y Sorensen B Irwin ML Morgan A Rudolph RE Surawicz C Lampe JW Ayub K Potter JD Effect of a 12-month exercise intervention on patterns of cellular proliferation in colonic crypts: a randomized controlled trial Cancer Epidemiol Biomarkers Prev 2006 15 9 1588 1597 10.1158/1055-9965.EPI-06-0223 16985018

[B65] McTiernan A Tworoger S Ulrich C Yasui Y Irwin M Rajan K Sorensen B Rudolph R Bowen D Stanczyk F Effect of exercise on serum estrogens in postmenopausal women: a 12-month randomized clinical trial Cancer Res 2004 64 2923 2928 10.1158/0008-5472.CAN-03-3393 15087413

[B66] Monninkhof EM Velthuis MJ Peeters PHM Twisk JWR Schuit AJ Effect of exercise on postmenopausal sex hormone levels and role of body fat: a randomized controlled trial J Clin Oncol 2009 27 27 4492 4499 10.1200/JCO.2008.19.7459 19687339

[B67] McTiernan A Tworoger S Rajan K Yasui Y Sorensen B Ulrich C Chubak J Stanczyk F Bowen D Irwin M Effect of exercise on serum androgens in postmenopausal women: a 12-month randomized clinical trial Cancer Epidemiol Biomarkers Prev 2004 13 7 1099 1105 15247119

[B68] Frank L Sorensen B Yasui Y Tworoger S Schwartz R Ulrich C Irwin M Rudolph R Rajan K Stanczyk F Effects of exercise on metabolic risk variables in overweight postmenopausal women: a randomized clinical trial Obes Res 2005 13 3 615 625 10.1038/oby.2005.66 15833948

[B69] Atkinson C Lampe JW Tworoger S Ulrich C Bowen D Irwin M Schwartz R Rajan K Yasui Y Potter J Effects of a moderate intensity exercise intervention on estrogen metabolism in postmenopausal women Cancer Epidemiol Biomarkers Prev 2004 13 5 868 874 15159321

[B70] McTiernan A Sorensen B Yasui Y Tworoger S Ulrich C Irwin M Rudolph R Stanczyk F Schwartz R Potter J No effect of exercise on insulin-like growth factor 1 and insulin-like growth factor binding protein 3 in postmenopausal women: a 12-month randomized clinical trial Cancer Epidemiol Biomarkers Prev 2005 14 4 1020 1021 10.1158/1055-9965.EPI-04-0834 15824183

[B71] Friedenreich CM Woolcott CG McTiernan A Ballard-Barbash R Brant RF Stanczyk FZ Terry T Boyd NF Yaffe MJ Irwin ML Alberta physical activity and breast cancer prevention trial: sex hormone changes in a year-long exercise intervention among postmenopausal women J Clin Oncol 2010 28 9 1458 1466 10.1200/JCO.2009.24.9557 20159820 PMC2849767

